# Right inferior frontal gyrus activation as a neural marker of successful lying

**DOI:** 10.3389/fnhum.2013.00616

**Published:** 2013-10-03

**Authors:** Oshin Vartanian, Peter J. Kwantes, David R. Mandel, Fethi Bouak, Ann Nakashima, Ingrid Smith, Quan Lam

**Affiliations:** ^1^Defence Research and Development CanadaToronto, ON, Canada; ^2^Department of Psychology, University of Toronto–ScarboroughToronto, ON, Canada; ^3^School of Psychology, University of QueenslandBrisbane, QLD, Australia; ^4^Department of Psychology, York UniversityToronto, ON, Canada

**Keywords:** deception, lying, inhibition

## Abstract

There is evidence to suggest that successful lying necessitates cognitive effort. We tested this hypothesis by instructing participants to lie or tell the truth under conditions of high and low working memory (WM) load. The task required participants to register a response on 80 trials of identical structure within a 2 (WM Load: high, low) × 2 (Instruction: truth or lie) repeated-measures design. Participants were less accurate and responded more slowly when WM load was high, and also when they lied. High WM load activated the fronto-parietal WM network including dorsolateral prefrontal cortex (PFC), middle frontal gyrus, precuneus, and intraparietal cortex. Lying activated areas previously shown to underlie deception, including middle and superior frontal gyrus and precuneus. Critically, successful lying in the high vs. low WM load condition was associated with longer response latency, and it activated the right inferior frontal gyrus—a key brain region regulating inhibition. The same pattern of activation in the inferior frontal gyrus was absent when participants told the truth. These findings demonstrate that lying under high cognitive load places a burden on inhibition, and that the right inferior frontal gyrus may provide a neural marker for successful lying.

A substantial body of behavioral evidence—collected both in the psychological laboratory as well as during police interviews—suggests that lying requires effort (Vrij et al., 2006, 2010). Given this observation, one potential strategy for catching a liar or detecting a lie would be to increase a suspect's cognitive load. To the extent that limited cognitive resources—including working memory (WM) and executive functions—are depleted, so is their availability to aid a liar to maintain a lie (see Vrij et al., 2010). Indeed, previous studies have demonstrated that a number of methodologies known to increase cognitive load are effective in helping to detect lies, including requiring subjects to maintain continuous eye contact (Beattie, 1981), asking questions that are irrelevant to some focal event (Quas et al., 2007), and instructing suspects to recall events in reverse order (Vrij et al., 2008). The present experiment was designed to test the hypothesis that a *direct* manipulation of WM load based on a variation of Sternberg's (1966) classic short-term memory paradigm will achieve the same result. Specifically, it will be more effortful to lie successfully when WM load is high compared to when WM load is low—as measured by an increase in response time (RT). Compared to previous approaches, the most salient feature of this technique is that the manipulation of WM load is non-verbal, and it can be implemented with ease on a trial-by-trial basis.

However, it has also been shown that the exertion of effort while lying could have multiple sources such as, the formulation of a lie, lie activation, self-monitoring of behavior, monitoring of the interviewer's behavior, truth suppression, and the implementation of reminders to lie (Vrij et al., 2010). This means that in addition to measures of cognitive effort, additional metrics are necessary to identify the source of the effort. One type of evidence that can be gainfully employed for this purpose is brain activation data, although the utility of brain imaging data depends on the specificity of the cognitive processes associated with the activated regions (Poldrack, 2006). In the context of the present study, the cognitive process that we were particularly interested in was inhibition, and its widely accepted role in truth suppression (e.g., Langleben et al., 2002). To determine whether the added burden on inhibition contributes to the increased effort while lying, we turned to data collected in the functional magnetic resonance imaging (fMRI) scanner. At the neural level, inhibition has been shown to be reliably correlated with activation in the inferior frontal gyrus (Aron et al., 2004), bolstered further by neuropsychological evidence demonstrating that persons with damage to this region are impaired at inhibition tasks (Aron et al., 2003). This evidence points to the inferior frontal gyrus as a likely candidate region for regulating inhibition during lying.

Vartanian et al. (2012) recently demonstrated that lying is correlated with increased activation in the WM network. They found that the inferior frontal gyrus was activated more in successful liars than in less-skilled ones. Based on scores taken from one condition of the task in which high variability in performance was observed, an independent samples *t*-test between good and poor liars revealed a significant difference in activation exclusive to the right inferior frontal gyrus (BA 44). Furthermore, a regression in which lying accuracy was regressed onto activation in the right inferior frontal gyrus demonstrated that activation in the right inferior frontal gyrus was a reliable predictor of lying accuracy, accounting for 29% of the observed variance in performance. The result suggests that individual differences in people‘s ability to supress the truth (as measured by activity in the right inferior frontal gyrus) is an important predictor of lying skill.

Extending from the approach employed by Vartanian et al. (2012), and based on Vrij et al.'s (2010) conjecture about how taxing cognitive load might help identify liars, we examined how the inferior frontal gyrus might be activated when participants lied successfully under high and low WM load, and compared it to when they were instructed to tell the truth under the same WM load conditions. Under instructions to tell the truth, participants do not need to suppress a truthful response. Without the need to supress a response, we did not expect an increase in WM load to have a strong impact on the activation of the inferior frontal gyrus. On the other hand, to the extent that depleting limited WM's resources increases inhibitory workload (Vrij et al., 2010), we predicted that the inferior frontal gyrus would experience substantially higher activation when participants lied under a high WM load than when lies were committed under low WM load.

## Materials and method

### Participants

This research proposal was approved by DRDC's Human Research Ethics Committee and Sunnybrook Health Sciences Centre's Research Ethics Board. The participants were 15 neurologically healthy right-handed volunteers (1/3 female, age range 19–48 years) with normal or corrected-to-normal vision.

### Stimuli and procedure

**Figure 1:**
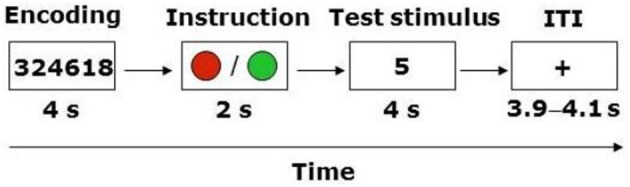
**Trial structure**. ITI, inter-trial-interval. ITI varied between 3900, 4000, and 4100 ms.

The task required participants to register a response on 80 trials of identical structure within a 2 (WM load, high or low) × 2 (Instruction: truth or lie) repeated-measures design (Figure 1). Trials involving the instruction to lie were distributed equally among match and no-match stimuli, resulting in 20 trials in each of the four conditions. The trial structure involved a modification of Sternberg's (1966) classic short-term memory paradigm, wherein participants are presented with a sequence of symbols (e.g., letters or digits) that must be encoded into memory, followed after a delay with the presentation of a test stimulus (i.e., a letter or a digit). The participant's task is to decide whether the test stimulus matches one of the symbols in the sequence presented earlier. The standard finding from that literature indicates that there is a linear relationship between the mean reaction time (RT) to make this decision and the length of the sequence. In the present experiment, each trial began with the presentation of a four- or six-digit string for 4 s. Participants were instructed to encode this string into memory. At the end of the trial the participant was presented with a test stimulus (i.e., a digit), and had to decide whether it matched one of the digits in the string within a 4 s response window. The variation in the length of the sequence (i.e., four vs. six digits) represented our WM manipulation. Notably, however, immediately following the presentation of the digit string participants were presented with a cue for 2 s that instructed them to either report truthfully or to lie about whether the test stimulus matched one of the digits in the string. In the truth condition the cue appeared as a green circle, whereas in the lie condition it appeared as a red circle.

Thus, the total duration of each trial was 10 s, and successive trials were interspersed with a fixation point with variable inter-trial interval (ITI, 3900, 4000, or 4100 ms averaged at 4 s across all trials). Participants recorded their responses using an MRI-compatible keypad that had separate keys labeled “match” and “mismatch.” Match and mismatch responses were registered using the index and middle finger of the same hand. The hand used to enter responses as well as the keys (for match and mismatch) were counterbalanced across participants. In the scanner, the 80 trials were presented in a single run. The order of trials was randomized for each participant. The duration of the task was 18 min and 40 s (80 trials × 14 s). Prior to entry into the scanner participants completed 10 practice trials to familiarize themselves with the task.

### fMRI acquisition and analysis

A 3-Tesla MR scanner with an 8-channel head coil (Discovery MR750, 22.0 software, GE Healthcare, Waukesha, WI) was used to acquire T1 anatomical volume images (0.86 × 0.86 × 1.0 mm voxels). For functional imaging, T2^*^-weighted gradient echo spiral-in/out acquisitions were used to produce 26 contiguous 5 mm thick axial slices [repetition time (TR) = 2000 ms; echo time (TE) = 30 ms; flip angle (FA) = 70°; field of view (FOV) = 200 mm; 64 × 64 matrix; voxel dimensions = 3.1 × 3.1 × 5.0 mm], positioned to cover the whole brain. The spiral sequence was acquired sequentially. The first five volumes were discarded to allow for T1 equilibration effects, leaving 560 volumes for analysis.

Data were analyzed using Statistical Parametric Mapping (SPM8). Head movement was less than 2 mm in all cases. All functional volumes were spatially realigned to the first volume. A mean image created from realigned volumes was spatially normalized to the Montreal Neurological Institute's echo-planar imaging (MNI EPI) brain template using non-linear basis functions. The derived spatial transformation was applied to the realigned T2^*^ volumes, and spatially smoothed with an 8 mm full-width half-maximum (FWHM) isotropic Gaussian kernel. Time series across each voxel were high-pass filtered with a cut-off of 128 s, using cosine functions to remove section-specific low frequency drifts in the BOLD signal. Condition effects at each voxel were estimated according to the GLM and regionally specific effects compared using linear contrasts. The BOLD signal was modeled as a box-car, convolved with a canonical hemodynamic response function. Each contrast produced a statistical parametric map consisting of voxels where the *z*-statistic was significant at *p* < 0.001. Reported activations survived voxel-level intensity threshold of *p* < 0.001 (uncorrected for multiple comparisons) at the voxel level and *p* < 0.05 (uncorrected for multiple comparisons) at the cluster level using a random-effects model.

## Results

### Behavioral

Mean correct RT and percent correct for each condition are shown in Table 1. Mean correct RT across all conditions was 1452 ms (*SEM* = 80). Skewness and kurtosis of the RT distribution did not deviate from normality (both *p*s > 0.05). A WM load × Instruction repeated-measures ANOVA showed the predicted main effect for WM such that RT was longer in the high load condition than in the low load condition, *F*_(1, 14)_ = 15.53, *p* < 0.001, partial η^2^ = 0.53. As well, we observed the predicted main effect for Instruction in which RT was longer in the lie condition than in the truth condition, *F*_(1, 14)_ = 55.03, *p* < 0.001, partial η^2^ = 0.80 (Table 1). The interaction between WM Load and Instruction was not reliable, *F*_(1, 14)_ = 0.45, *p* = 0.51, partial η^2^ = 0.03.

**Table 1:** **Response time (in ms) and accuracy (expressed as a percentage) with standard errors (*SE*) in brackets for each condition**.

**Instructions**				
**Lie**			**Truth**	
WM load	RT (*SE*)	Accuracy (*SE*)	RT (*SE*)	Accuracy (*SE*)
High	1719 (99)	91 (0.08)	1332 (84)	94 (0.06)
Low	1555 (91)	94 (0.01)	1203 (71)	98 (0.01)

Mean accuracy across all conditions was 94.4% (*SEM* = 0.01). Skewness and kurtosis of the accuracy distribution did not deviate from normality (both *p*s > 0.05). A WM load × Instruction repeated-measures ANOVA showed the predicted main effect for WM load: accuracy was lower in the high load condition than in the low load condition, *F*_(1, 14)_ = 8.61, *p* < 0.05, partial η^2^ = 0.38 As well, we observed the predicted main effect for instruction: accuracy was lower in the lie condition than in the truth condition, *F*_(1, 14)_ = 7.32, *p* < 0.05, partial η^2^ = 0.34 (Table 1). The interaction between WM Load and Instruction was not reliable, *F*_(1, 14)_ = 0.45, *p* = 0.56, partial η^2^ = 0.03.

### fMRI

Using an event-related design, at the first level of analysis (i.e., subject level in SPM8) we specified regressors corresponding to the four conditions, as well as ITI and motor response. Although incorporated into the design, ITI and motor response were modeled out of the analyses by assigning null weights to their regressors. Given the main effect of WM load, we investigated the direct contrast of high vs. low WM load. This demonstrated significant activation in the middle frontal gyrus, precuneus, intraparietal sulcus, supplementary motor area, caudate, dorsolateral PFC, and cerebellum (Table 2). This pattern is consistent with the well-established role of the frontoparietal system in WM, and indeed specifically as observed within the Sternberg paradigm (Zarahn et al., 2006).

**Table 2:** **Activated regions corresponding to reported contrasts**.

	**BA**	**L**	**Z**	***x***	***y***	***z***
**HI WM LOAD–LOW WM LOAD**						
Middle frontal gyrus	6	l	5.28	−50	8	38
	6	r	4.08	34	0	26
Precuneus	7	l	4.95	−4	−74	60
Intraparietal sulcus	40	l	4.39	−30	−58	44
Cerebellum	−	l	4.31	−8	−90	−24
Supplementary motor area	6	l	3.92	−4	−4	60
Caudate	−	r	3.92	12	−8	20
Dorsolateral PFC	46	r	3.74	48	22	30
**LYING–TRUTHFUL REPORTING**						
Middle frontal gyrus	6	l	5.18	−38	16	46
Superior frontal gyrus	6	l	4.67	−4	32	50
Precuneus	7	l	4.19	−44	−50	46
	7	r	3.84	42	−60	42
Middle temporal gyrus	21	r	3.65	60	−32	−10

Regions are designated using MNI coordinates; BA indicates Brodmann area; L indicates laterality; l and r indicate left and right hemispheres, respectively; Z indicates z–score.

Next, given the main effect of Instruction, we investigated the direct contrast of lying–truthful reporting. This demonstrated significant activation in middle and superior frontal gyri, bilateral precuneus, and middle temporal gyrus (Table 2). The middle frontal gyrus and precuneus were activated in the lying–truthful reporting contrast in Vartanian et al. (2012) and elsewhere (e.g., Ganis et al., 2003).

**Figure 2:**
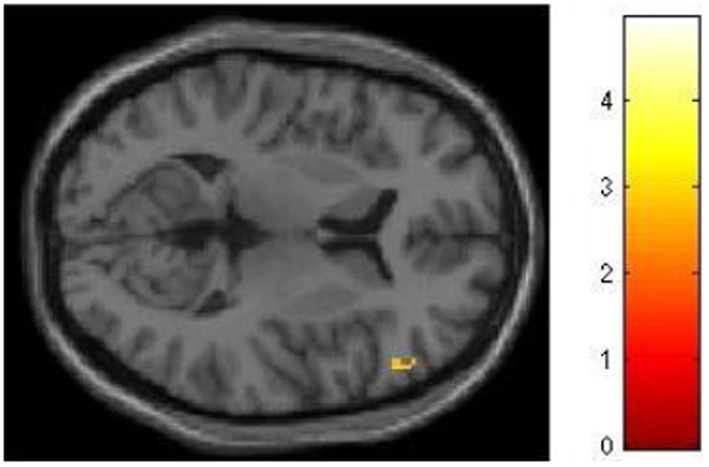
**The right inferior frontal gyrus is activated more for successful lying under high WM load than low WM load**. SPM rendered into standard stereotactic space and superimposed on to transverse MRI in standard space. The bar graph represents the strength of the activation (*T*–score).

**Figure 3:**
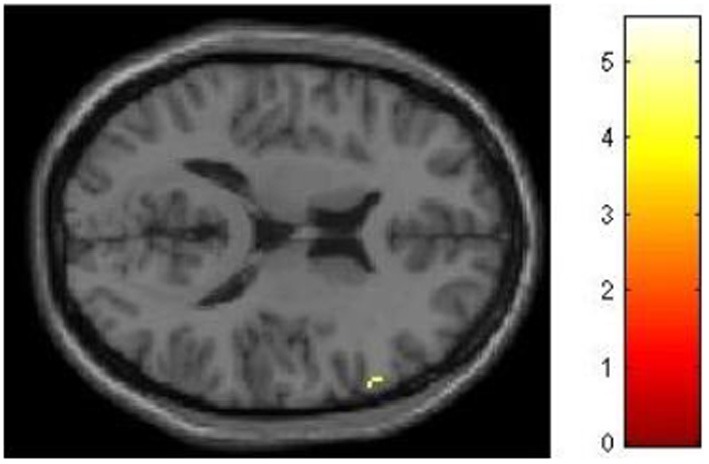
**High working memory load activates the right inferior frontal gyrus more when lying successfully than when telling the truth successfully**. SPM rendered into standard stereotactic space and superimposed on to transverse MRI in standard space. The bar graph represents the strength of the activation (*T*–score).

We had hypothesized that successful lying would place greater demands on inhibition under high WM load than under low WM load, but that truthful reporting would not place similar demands on inhibition under the same conditions. To test this hypothesis we selected those trials for which an accurate response was collected and compared responses under high and low WM load conditions. We did a direct contrast of the high vs. low WM load condition, but akin to what Vartanian et al. (2012) did, we selected for our Small Volume Correction in SPM8 a spherical region of interest (ROI) in the right inferior lateral prefrontal cortex (PFC) (coordinates of the center of mass *x* = 51, *y* = 21, *z* = 12) with a radius of 10 mm. This specific ROI was selected from Goel and Dolan (2003) in which it was associated with inhibition in reasoning. The same ROI was used by De Neys et al. (2006) as the ROI for inhibition in decision making. As shown in Figure 2, the high–low WM load contrast revealed significant activation in the right inferior frontal gyrus under instructions to lie (BA 45) (54, 30, 8, *z* = 2.49, *p* = 0.006). This activation was not present under instructions to tell the truth. Critically, an interaction analysis revealed a significantly greater difference between high and low WM load under instructions to lie than to tell the truth in two areas also located in the inferior frontal gyrus (BA 45) (62, 20, 16, *z* = 3.43, *p* < 0.001; 54, 26, 10, *z* = 3.13, *p* < 0.001) (Figure 3). In other words, high WM load activated the right inferior frontal gyrus more when lying successfully than when telling the truth successfully.

## Discussion

Our results are consistent with previous work reporting increased RT in response to increased WM load (e.g., Sternberg, 1966) and the requirement to lie (e.g., Holden and Hibbs, 1995). Our findings and interpretation are also consistent with Vrij et al.'s (2010) hypothesis that one strategy to detect deception is the placement of greater cognitive load on a suspect. More critically, however, our neurological data showed that when WM capacity is depleted, inhibitory workload (as measured by the BOLD signal) is increased specifically for those trials on which participants were required to suppress the truth and respond with a lie.

Several papers in the neuroscience literature have demonstrated that deception activates neural systems underlying WM and executive functions (for reviews see Spence et al., 2004; Sip et al., 2008; Abe, 2009, 2011). The involvement of the PFC has been a recurrent theme, particularly because of its known role in inhibiting behavior, which in the case of lying involves the suppression of truthful responses. Our results contribute to this literature by demonstrating the role of the right inferior frontal gyrus in successful lying in the high vs. low WM load condition^1^. Specifically, we have shown that WM load differentially impacts brain function in right inferior frontal gyrus under instructions to lie, but not under instructions to tell the truth. We postulate that the particular role of the right inferior frontal gyrus during a lie is to suppress the truthful response, and that suppression requires more effort when WM is taxed. This interpretation is also consistent with the RT difference observed between the high and low load conditions when participants were instructed to lie.

One could argue that our participants might have adopted a task-switching strategy under instructions to lie. In other words, the instruction to lie could invoke a switch in the mapping between stimulus and response, and as such reveal little about the participants' intention to lie. Our survey of the recent task-switching literature suggests that such an interpretation is unlikely. Specifically, in several of the papers we reviewed (e.g., Meiran, 2000; Vu and Proctor, 2004; Crone et al., 2006), when participants were cued to indicate the mapping to be applied to the stimulus, the cost in terms of response time for switching tended to be around 100 ms. If our lying manipulation triggered a strategy in which participants simply reassigned the stimulus-response mapping depending on the cue, we should have witnessed a similar cost in response time. By contrast, our comparatively large RT cost (over 300 ms) suggests more effortful processing of the stimulus, and that the activation exhibited in the right inferior frontal gyrus is more likely associated with lying rather than simple task switching.

It has been noted before that an important limitation of studies of lie detection involves the use of experimental designs in which participants were instructed to lie on demand (see Sip et al., 2008). This criticism raises important concerns about the ecological validity of the employed methodologies and by extension, empirical findings. Two recent studies have challenged this criticism by enabling participants to engage in spontaneous lying. Greene and Paxton (2009) instructed their participants to predict the outcomes of computerized coin flips while they were being scanned with fMRI. Correct predictions were rewarded by monetary gain. Importantly, in some trials participants were rewarded based on self-reported accuracy. This allowed them to gain money dishonestly by lying about the accuracy of their predictions. Indeed, given this opportunity many participants behaved dishonestly by lying about their predictions, assessed by improbably high levels of deviation from chance (i.e., 50% for coin flips). Their fMRI results revealed that lying was associated with neural activity in anterior cingulate cortex (ACC), dorsolateral PFC and the inferior frontal gyrus. In addition, activation in these regions was also associated with individual differences in the frequency of lying. This individual-differences result is particularly interesting because it links a tendency to engage in lying to the same region that Vartanian et al. (2012) found to predict lying skill—the right inferior frontal gyrus.

In a more recent study, Ding et al. (2013) used near-infrared spectroscopy (fNIRS) to study spontaneous deception. NIRS is a non-invasive imaging method that allows *in-vivo* photometric measurement of changes in the concentrations of oxygenated and deoxygenated hemoglobin in the cortex, and can thus be used to characterize physiological blood oxygenation changes in relation to cognitive tasks. The participants' task was to predict, on each trial, the side of the screen in which a coin would appear. Participants put each of their hands in one of two drawers of a desk (so their hand movements would not be directly visible to the experimenter). Participants made their predictions by moving their hand corresponding to the predicted side. Following the presentation of the coin on the screen, a message on the screen asked them whether they had guessed the location of the coin correctly. However, unbeknownst to the participants, the experimenters had installed hidden cameras inside the drawers to record the movement of each participant's hands. This enabled the experimenters to determine, on a trial-by-trial basis, whether the participants had engaged in spontaneous deception. The results demonstrated that lying was correlated with increased activity in left superior frontal gyrus (BA 6)—the area also activated in Vartanian et al. (2012) and the present study in the lying–truthful contrast. Thus, the studies by Greene and Paxton (2009) and Ding et al. (2013) demonstrate that the PFC plays a role in deception—regardless of whether it occurs spontaneously or is triggered on demand. Nevertheless, given that standard fMRI activation patterns are expressed as subtractions, not only is the choice of an appropriate control condition vis-à-vis lying critical for meaningful interpretation of results (Friston et al., 1996), but also vital for a meaningful comparison of the findings reported across laboratories.

Based on recent theoretical and methodological advances in the neuroscience of deception, it would appear that neuroimaging has the potential to eventually develop into a useful part of the forensic toolkit for lie detection (for reviews see Abe, 2009, 2011). However, important questions remain unanswered. For example, because neuroimaging studies are correlational, they cannot definitively determine the necessity of any brain region for deception. Evidence that can determine necessity is provided by loss-of-function studies that investigate permanent inability to lie as a function of neuropsychological impairment, or a transient inability to lie due to “temporary lesions” instantiated using transcranial magnetic stimulation (TMS) or transcranial direct current stimulation (tDCS) (for a review see Luber et al., 2009). Unfortunately, evidence from loss-of-function studies regarding the role of PFC in deception has been inconsistent. For example, Luber and colleagues, using a variant of the Guilty Knowledge Test [adapted from Langleben et al. (2002, 2005)], applied TMS pulses to left DLPFC and parietal cortex to disrupt the neural circuitry shown to be correlated with the formation of deceptive responses. The results demonstrated that TMS pulses applied exclusively to the parietal cortex increased RT by 20%, whereas the same stimulation applied to left DLPFC alone had no effect on RT. These results cast doubt on the necessity of PFC for the formation of lies (see also Verschuere et al., 2012). On the other hand, Priori et al. (2008) found that applying anodal tDCS to bilateral DLPFC did increase RT for denial lies. The inconsistency suggests that continued study is needed to determine precisely the conditions under which PFC and its subregions necessarily contribute to specific aspects of deception.

In addition, there does not appear to be an activation pattern that is unique to lying or deception (Wolpe et al., 2005; Appelbaum, 2007; Sip et al., 2008). Rather, as is the case with other higher-order mental processes such as reasoning and decision making (Goel, 2007; Frank et al., 2009), lying and deception appear to be built on multiple neural systems that are differentially activated as a function of task and contextual demands. In the case of lying and deception those processes include, among others, WM, error monitoring, response selection, and target detection (Hester et al., 2004; Huettel and McCarthy, 2004; Zarahn et al., 2006). This makes the use of fMRI for lie detection in forensic and legal settings challenging, given that practitioners in applied settings will be unable to make clear-cut judgments of guilt based on fMRI data alone. However, neuroimaging data could comprise one of many components of a broader arsenal for detecting deception. For example, according to the “information gathering” approach to lie detection, interviewers are instructed to focus on gathering verbal information from suspects that can be subsequently checked for inconsistencies against available evidence (Vrij et al., 2010). The approach is predicated on not focusing on a single cue, but rather collecting and cross-referencing their consistency. By providing neural information, neuroimaging evidence can contribute to the forensic decision-making apparatus in this context. This componential approach was reinforced in a recent report by the National Academy of Sciences (2008). We suggest that a broad set of metrics that combines verbal, non-verbal, and neural data provides the most promising framework for lie detection in the lab and elsewhere.

### Conflict of interest statement

The authors declare that the research was conducted in the absence of any commercial or financial relationships that could be construed as a potential conflict of interest.

## Footnotes

^1^Despite the low z -score in relation to the simple main effect of WM load under instructions to lie, the results were consistent with the interaction analysis that also revealed significantly greater difference in activation in right inferior frontal gyrus between high and low WM load under instructions to successfully lie than to tell the truth.
